# An AMP-activated protein kinase complex with two distinctive alpha subunits is involved in nutritional stress responses in *Trypanosoma cruzi*

**DOI:** 10.1371/journal.pntd.0009435

**Published:** 2021-05-24

**Authors:** Tamara Sternlieb, Alejandra C. Schoijet, Patricio D. Genta, Salomé C. Vilchez Larrea, Guillermo D. Alonso

**Affiliations:** 1 Laboratorio de señalización y mecanismos adaptativos en tripanosomátidos, Instituto de Investigaciones en Ingeniería Genética y Biología Molecular “Dr. Héctor N. Torres”, Buenos Aires, Argentina; 2 Departamento de Química Biológica, Facultad de Ciencias Exactas y Naturales, Universidad de Buenos Aires, Buenos Aires, Argentina; 3 Laboratorio de ADP - Ribósidos y Enfermedades Parasitarias, Instituto de Investigaciones en Ingeniería Genética y Biología Molecular “Dr. Héctor N. Torres”, Buenos Aires, Argentina; 4 Departamento de Fisiología, Biología Molecular y Celular, Facultad de Ciencias Exactas y Naturales, Universidad de Buenos Aires, Buenos Aires, Argentina; University of Texas at El Paso, UNITED STATES

## Abstract

*Trypanosoma cruzi*, the etiological agent of Chagas disease, has a digenetic life cycle. In its passage from the insect vector to the mammalian host, and vice versa, it must be prepared to cope with abrupt changes in environmental conditions, such as carbon source, pH, temperature and osmolarity, in order to survive. Sensing and signaling pathways that allow the parasite to adapt, have unique characteristics with respect to their hosts and other free-living organisms. Many of the canonical proteins involved in these transduction pathways have not yet been found in the genomes of these parasites because they present divergences either at the functional, structural and/or protein sequence level. All of this makes these pathways promising targets for therapeutic drugs. The AMP-activated protein kinase (AMPK) is a serine/threonine kinase activated by environmental stresses such as osmotic stress, hypoxia, ischaemia and exercise that results in reduction of ATP and increase of AMP levels. Thus, AMPK is regarded as a fuel gauge, functioning both as a nutrient and an energy sensor, to maintain energy homeostasis and, eventually, to protect cells from death by nutrient starvation. In the present study we report the characterization of AMPK complexes for the first time in *T*. *cruzi* and propose the function of TcAMPK as a novel regulator of nutritional stress in epimastigote forms. We show that there is phosphotransferase activity specific for SAMS peptide in epimastigotes extracts, which is inhibited by Compound C and is modulated by carbon source availability. In addition, TcAMPKα2 subunit has an unprecedented functional substitution (Ser x Thr) at the activation loop and its overexpression in epimastigotes led to higher autophagic activity during prolonged nutritional stress. Moreover, the over-expression of the catalytic subunits resulted in antagonistic phenotypes associated with proliferation. Together, these results point to a role of TcAMPK in autophagy and nutrient sensing, key processes for the survival of trypanosomatids and for its life cycle progression.

## Author summary

Chagas disease is caused by the protozoan parasite *Trypanosoma cruzi*. This silent illness is endemic in Latin-American countries and is conventionally transmitted to humans by insects from the Reduviidae family. In its passage from the insect vector to the mammalian host, and vice versa, the parasite must overcome abrupt changes in environmental conditions in order to survive.

The AMP-activated protein kinase (AMPK) is a serine/threonine protein kinase activated by environmental stresses that cause imbalances of the intracellular AMP:ATP ratios. Thus, AMPK is regarded as a “fuel gauge”, functioning both as a nutrient and an energy sensor to help maintain energy homeostasis and protect cells from death by nutrient starvation.

In the present study we report the characterization of AMPK complexes for the first time in *T*. *cruzi* and describe the function of AMPK as a novel regulator of nutritional stress in epimastigote forms. We demonstrate that this complex possesses specific AMPK kinase activity, is inhibited by Compound C and is modulated by carbon source availability.

Together, these results point to a role of AMPK in autophagy and nutrient sensing, key processes for the survival of this parasite and for its life cycle progression.

## Introduction

Chagas disease, also known as American trypanosomiasis, is a potentially life-threatening illness endemic in Latin-American countries. This silent illness is conventionally caused when insects from the *Reduviidae* family transmit the protozoan parasite *Trypanosoma cruzi* to humans. It is also included by the World Health Organization in the 20 groups of Neglected Tropical Diseases, collectively affecting about 2 billion people worldwide [1,2].

Very few drugs are annually developed for treatment of NTDs [3] and so, the development of new technologies, higher government and industrial involvement and more scientists committed to basic investigation, are key to find new alternatives. In this sense, signal transduction pathways in trypanosomatids could be considered as an Achilles´ heel, since they are essential to recognize environmental fluctuations and allow these parasites to respond accurately through cellular changes. On this matter, advances in knowledge on the differences of these pathways between the parasite and mammalian cells might allow the identification of rational targets for the development of safe and more effective drugs for Chagas disease treatment [3].

*Trypanosoma cruzi* has a complex life cycle involving four main morphogenetic stages. Briefly, the life cycle of this parasite involves two intermediate hosts (triatomine insects and mammals, including man) and four well-defined morphological and functional developmental stages: epimastigotes, metacyclic trypomastigotes, amastigotes and bloodstream trypomastigotes. The epimastigote forms replicate in the midgut of the insect host and develop into non-replicative metacyclic trypomastigote forms. When the insects feed on blood, they release in their excreta metacyclic trypomastigotes that penetrate the body of the mammalian host through the wound and are able to invade cells. Within the host cells, the parasite differentiates into the replicative amastigote form. After multiplication, the amastigotes differentiate into bloodstream trypomastigotes that are released into the circulatory system, infecting new cells. Signal transduction largely controls the manner in which cells respond to stimuli and is essential for the progression of trypanosomatids’ life cycles.

In addition, these parasites are also of biological interest since they possess structures and organelles that are not found in mammalian cells, such as glycosomes, which are involved in glucose metabolism, and a single ramified tubular mitochondrion with a unique network of condensed concatenated circular mDNA called the kinetoplast. Therefore, understanding of the unique pathways in this pathogen may lead to the development of novel therapeutic agents. In particular, targeting metabolic pathways in the parasite for rational drug design represents a promising research field.

Fluctuations in environmental conditions cause cellular stress, which in turn force the cells to remodel their metabolism to cope and survive. This is achieved through several intracellular messengers, which transduce the extracellular signal to the intracellular environment. Cells with a sufficient energy supply maintain a balance between the adenosine phosphate species of 10:1 for ATP:ADP and 100:1 for ATP:AMP, thanks to the interconversion between them, executed by ATPases, ATP synthases and Adenylate Kinases [4]. From the nature of these enzymatic reactions, when the energy status is affected and ATP diminishes, the AMP:ATP ratio increases as the square of the ADP:ATP ratio. This explains why most energy sensing mechanisms in the cell depend on AMP detection and activation. One of these mechanisms is the AMP-activated protein kinase (AMPK) complex.

The cellular energy homeostasis can be affected by different kinds of stresses, such as nutritional, oxidative and heat shock, amongst others. Enzymes like the AMPK can respond in a very sensitive manner to the imbalances caused by these stresses and thus shift cellular anabolic activity to a more catabolic one, which replenishes the ATP levels [5].

The AMPK complex exists as a heterotrimer, composed of an alpha catalytic subunit and two regulatory subunits, beta and gamma. The α subunit contains a kinase domain as well as a regulatory domain that inhibits the enzyme in the absence of AMP [6]. The β subunit acts as a scaffold for the other components while also modulating the localization of the complex [7–9], and the γ subunit is involved in AMP binding [10]. With very few exceptions, every eukaryotic organism expresses at least one AMPK complex. Notably, the eukaryotes that lack AMPK orthologues are parasites that are exclusively intracellular or most of their life elapses inside their host’s cells [11]. The amino acid sequence of the subunits varies between different organisms but conserves a domain architecture that allows the execution of ancient functions. Yet, small mutations in some codons also led to neofunctionalization of the orthologs and paralogs, diversifying the role of this complex between organisms and tissues [12–14]. The AMPK yeast ortholog, SNF1, is known for its role in the derepression of glucose-repressed genes [15]. In higher eukaryotes, AMPK phosphorylates enzymes of the isoprenoid synthesis and fatty acid synthesis pathways to inhibit these metabolisms and switch the cell to the use of stored or alternative carbon sources, such as fatty acids [16]. AMPK can also phosphorylate transcription factors, inducing the expression of proteins involved in several stress response pathways. Through all these functions, this complex has been established as an essential regulatory hub for the cell.

Parasitic organisms fully depend on the environmental signals in their hosts to progress in their life cycle. The rapid passage through these environments also exerts several kinds of stresses, such as wide changes in osmolarity, pH, temperature and nutrient availability, that parasites must overcome to survive. Molecular stress sensors, including AMPK, can play key roles in these processes. Recent studies have reported that *T*. *brucei* expresses an AMPK complex (TbAMPK) with two possible catalytic α subunits. TbAMPK is involved in the expression of membrane proteins in response to nutritional stress and in the transition from the slender bloodstream form to the quiescent stumpy [17,18]. Also, Saldivia et. al. demonstrated that this complex is responsible for the growth arrest observed when parasites are treated with AMP analogs. TbAMPK complex containing the α1 subunit is also capable of responding to AMP:ATP imbalances produced by mitochondrial depolarization and, once activated, it phosphorylates proteins in the glycosomes, the organelles nucleating the first steps of glycolysis in these organisms [19]. However, some of the conventional roles of the AMPK complexes may not be conserved in the trypanosomes. Autophagy, a process that allows cells to consume their own components to provide nutrients and overcome stress, and which involves proteins activated by AMPK phosphorylation in many organisms [20–22], does not appear to depend on AMPK activation in *T*. *brucei* [23]. All these recent discoveries point to novel roles of AMPK in trypanosomatids, associated with their complex life cycle and ability to differentiate in response to environmental cues.

In this work, we identify the corresponding genes for the TcAMPK subunits, confirm the presence of a specific phosphotransferase activity on the SAMS substrate in the epimastigote stage, which is affected by nutritional stress. Finally, we show that the TcAMPK complex containing the α2 subunit is involved in autophagy when parasites are deprived of a carbon source, being this the first report where an AMPK complex from trypanosomatids shows to be involved in autophagy.

## Materials and methods

### Parasite cultures

*T*. *cruzi* epimastigotes of the CL Brener strain were cultured at 28°C for 7 days in liver infusion tryptose (LIT) medium (5 g/l liver infusion, 5 g/l bacto-tryptose, 68 mM NaCl, 5.3 mM KCl, 22 mM Na_2_HPO_4_, 0.2% (w/v) glucose, and 0.002% (w/v) hemin) supplemented with 10% (v/v) FCS, 100 U/ml penicillin and 100 mg/l streptomycin. Cell viability was assessed by direct microscopic examination. When assessing growth curves, doubling times were calculated as follows:

$$Td(h)=\frac{ln(2)}{slope\;(day\;3\;to\;day\;7)}$$

Where the slope between day 3 and 7 was obtained by a linear regression on the quantification of parasites in a Neubauer chamber over the number of days.

### Generation of TcAMPK-subunits overexpressing cell lines in *T*. *cruzi epimastigotes*

The full-length genes of every TcAMPK subunit were amplified using the following primers: for TcAMPKα1 TcAMPKα1-Fw-HindIII 5’-GGATCCATGAGTCAGAAGTTTGGCCCCTA-3’ and TcAMPKα1-Rv-pRIBOHA-XhoI 5’-CTCGAGTTAAGCGTAATCTGGAACATCGTATGGGTATTCGTCTGGTCCAAGAGAGGAA-3’. For TcAMPKα2 TcAMPKα2-Fw-HindIII 5’-AAGCTTATGCATTCCAGGCGGGATGTT-3’ and TcAMPKα2-Rv-pRIBOHA-XhoI 5’-CTCGAGTTAAGCGTAATCTGGAACATCGTATGGGTAACCCATTCGATGAACGAGCGTC-3’. For TcAMPKβ TcAMPKβ-Fw-HindIII 5’-GGTACCATGGGCCAACAAAATGCCAGGGA-3´ and TcAMPKβ-Rv-pRIBOHA-XhoI 5´-CTCGAGTTAAGCGTAATCTGGAACATCGTATGGGTACCCGTTCGGAGCTCCCATTCT-3’. For TcAMPKγ TcAMPKγ-Fw-HindIII 5’-AAGCTTATGCGTCGCACGAGTGCCTTTGC-3’ and TcAMPKγ-Rv-pRIBOHA-XhoI 5’-CTCGAGTTAAGCGTAATCTGGAACATCGTATGGGTATTTTTGTGCGTTGCCGTCATT-3’. The PCR products, now containing an hemagglutinin tag at their C-terminal end, were then cloned into pGEM-T Easy plasmid, the sequence identity was confirmed by DNA sequencing, and subcloned into the pRIBOTEX plasmid [24]. *T*. *cruzi* epimastigotes of CL Brener strain were transfected with the pRIBOTEX constructs as described previously [25]. Stable cell lines were achieved after 60 days of treatment with 500 μg/ml G418 (Gibco BRL, Carlsbad, CA) and the transgenic condition was confirmed by western blot analyses.

### Yeast transformation and functional complementation

Conditional *Saccharomyces cerevisiae* yeast mutant strains were generously provided by Dr. Martin C. Schmidt, from the University of Pittsburgh [26,27]. The same pGEM-T Easy constructs with each TcAMPK subunit were used to subclone into the p416 yeast expression plasmid. Yeasts were transformed following the “Quick and Dirty” protocol [28]. Briefly, a fresh yeast inoculum was mixed with 100 μl of freshly prepared transformation mix (200 μl of 2 M sterilized LiAc, 800 μl of sterilized 50% PEG-3350, 7.7 μl of 14 M 2-Mercaptoethanol), 3 μl of denaturalized salmon sperm DNA (10 mg/ml) and 1 μg of plasmid DNA or distilled water as control. The mix was incubated at 37°C for 30 min while mixing. After centrifugation at 3000 rpm for 5 min, the pellet was recovered and resuspended in 100 μl of sterile water. The transformed yeasts were plated on selective media (lacking uracil) and were grown for 16 to 72 h at 30°C. Individual colonies were tested for heterologous protein expression. A positive clone was grown overnight at 30°C in YPAD media (yeast extract 10 g/l, peptone 20 g/l, glucose 20 g/l, adenine sulfate 0.04 g/l) up to an OD_600_ of 2. Serial dilutions of the culture were plated on selective media without uracil (0.17% (w/v) yeast nitrogen base (without amino acids and ammonium sulphate) and 0.5% (w/v) ammonium sulphate, supplemented with the corresponding amino acid mixture) and containing either glucose (2% w/v) or raffinose (2% w/v) as the carbon source. Plates were incubated at 30°C between 3 to 5 days after which growth was photographed.

### Protein kinase assay

*In vitro* kinase reactions were developed in a final volume of 50 μl containing 0.02 mM [γ-^32^P]ATP (1 μCi per tube, Perkin Elmer, Massachusetts, USA), 50 mM Tris-HCl, pH 7.0, 0.1 mM NaCl, 0.1 mM EDTA, 0.5 mM dithiothreitol, 5 mM MgCl_2_ and 100 μM SAMS peptide (HMRSAMSGLHLVKRR, ab120182, Abcam) as AMPK substrate. The reaction was initiated by adding 50 μg of epimastigote protein extract and was incubated with shaking at 30°C for 10 min. As enzyme blank control, tubes where protein extract was replaced by lysis buffer were added. The reaction was stopped by immediate immersion in an ice bath and spotting on P81 Whatman filter paper. Unreacted ATP was removed washing the filter papers 3 times with 1% phosphoric acid for 7 min. After the final wash, the filters were quickly dried with ethanol, placed in polistor tubes with 2 ml of Ultima gold XR liquid scintillation cocktail (Perkin Elmer, Massachusetts, USA) and counted in a scintillation counter. AMPK activity was calculated as phosphorus incorporation subtracting the average counts per minute (cpm) of the enzyme blank controls from the average cpm of protein extract samples. For Dorsomorphin (CC) (#P5499, Sigma Aldrich, St. Louis, Missouri, United States or ab120843, Abcam, Cambridge, United Kingdom) treatment protein extracts were incubated in an ice bath with the 1 μM final concentration of the inhibitor for 10 min previous to the addition of the mix containing the SAMS.

### Cell extracts and western blotting

To obtain *T*. *cruzi* extracts, 10^8^ epimastigotes were harvested by centrifugation at 1500 g for 10 min and washed two times with phosphate-buffered saline (PBS). Cell pellets were then resuspended in lysis buffer (50 mM Tris-HCl buffer, pH 7.5; 14 mM 2-Mercaptoethanol, PMSF and E64 as proteases inhibitors and NaF and Na_2_VO_3_ as phosphatase inhibitors) and lysed by six cycles of freezing in liquid N_2_ and thawing at 4°C.

For western blotting analysis, proteins were solved in 10% (w/v) SDS-polyacrylamide gel electrophoresis as described by Laemmli (1970) and electrotransferred to Hybond-C membranes (Amersham Pharmacia Biotech, Piscataway, USA). The membranes were blocked with 5% (w/v) non-fat milk or 5% (w/v) BSA suspension in 0.05% TBS-Tween for at least 3 h. Blocked membranes were then incubated overnight with a 1:1000 dilution of Phospho-AMPKα (Thr172) (40H9) Rabbit mAb (#2535, Cell Signaling, Massachusetts, USA). Detection was carried out by incubating with a 1:5000 dilution of a goat anti-rabbit antibody conjugated to peroxidase (Sigma Aldrich). For tagged proteins detection, membranes were incubated for at least 1 h with a 1:1000 dilution of a high affinity anti-HA antibody from rat IgG1 (#11867423001, Roche Applied Science, Penzberg, Germany). Detection was carried out by incubating with a 1:4000 dilution of a rabbit anti-rat antibody conjugated to peroxidase (A5795, Sigma-Aldrich). A 1:20000 dilution of anti-α tubulin (B5-1-2) Mouse mAb (T5168, Sigma-Aldrich) was used to detect α tubulin as a loading control. The membranes were then developed with the ECL Plus Western blotting detection system (PerkinElmer Life Sciences, Massachusetts, USA).

As negative controls to check the expression of HA-tagged alpha subunits, either wild type parasites, parasites transfected with the empty vector or parasites transfected with another unrelated protein of different molecular weight were used.

For AICAR (ab120358, Abcam, Cambridge, United Kingdom) treatment, epimastigotes were incubated for 30 min with a final concentration of 1 mM AICAR in LIT media. For CC and AICAR simultaneous treatment, epimastigotes were incubated with both compounds (10 μM of CC) for 1 h.

When specified, aliquots of the same protein extract were separated and treated with 200 U of Lambda Phage Phosphatase (#P0753S, New England Biolabs, Ipswich, Massachusetts, United States), as specified by the manufacturer, for different time periods. The reaction was stopped by boiling in Laemmli buffer.

### Proteomic analysis

After 10% SDS-PAGE of epimastigotes protein extracts, the section of the gel between 70 kDa and 100 kDa was manually cut and preserved in an eppendorf tube at -80°C until delivery for mass spectrometry analysis. Mass spectrometry analysis was carried out at Centro de Estudios Químicos y Biológicos por Espectrometría de Masa (CEQUIBIEM), Argentina, in a Q Exactive HESI-Orbitrap coupled to a nano HPLC Easy-nLC 1000 (Thermo Scientific). Resulting reads were matched to the *T*. *cruzi* proteome available at the UNIPROT proteome database.

### Indirect immunofluorescence

For immunofluorescence, cells were fixed with 4% paraformaldehyde in PBS for 20 min. Next, the cells were washed twice in Dulbecco’s PBS, pH 7.2, adhered to poly-L-lysine-coated coverslips, and permeabilized for 10 min with 0.3% Triton X-100. Cells were incubated for 30 min with 25 mM ammonium chloride and washed again with PBS, after which they were blocked for 20 min in 3% bovine serum albumin in PBS, pH 8.0, and incubated for 1 h with rat anti-HA high affinity monoclonal antibodies (Roche Applied Science, Penzberg, Germany) at 1:500. Cells were then washed in 0.05% TBS-Tween buffer, incubated with the secondary antibody, anti-rat Alexa 546 conjugate at 1:500, and mounted with Vectashield (Vector Laboratories, California, USA) containing 5 mg/ml DAPI. Cells were observed in an Olympus BX41 fluorescence microscope and images were captured.

### Autophagy monitoring by Monodansylcadaverine incorporation

Epimastigotes in exponential phase (2x10^7^ parasites per ml) were washed with PBS twice and resuspended in fresh LIT medium as control or PBS for starvation. Cultures were incubated at 28°C for 17 h. Staining of autophagosomes with Monodansylcadaverine (MDC, #D4008, Sigma-Aldrich, St. Louis, Missouri, United States) was applied as in Munafó and Colombo (2001) [29]. Briefly, after 16 h starvation of epimastigotes, MDC was added at 0.05 mM final concentration and incubation proceeded for 1 h at 28°C. Epimastigotes were then washed two times with PBS and lysed in a buffer containing 10 mM Tris HCl pH 8.0 and 1% v/v Triton-X100. Lysates were distributed in triplicates in a 96 well plate and Ethidium bromide was added to the lysates at 0.2 μM per well. Fluorescence emission was measured at 380 nm excitation and 525 nm emission for MDC and 530 nm excitation and 590 nm emission for Ethidium bromide. Readings were quantified by fluorescence photometry in a Synergy HTX Multi-mode plate reader and normalization of the measures was calculated as MDC_525nm_/BrEt_590nm_ and expressed in arbitrary units.

## Results

### *In silico* identification of TcAMPK subunits and evolutionary analysis

**Fig 1:**
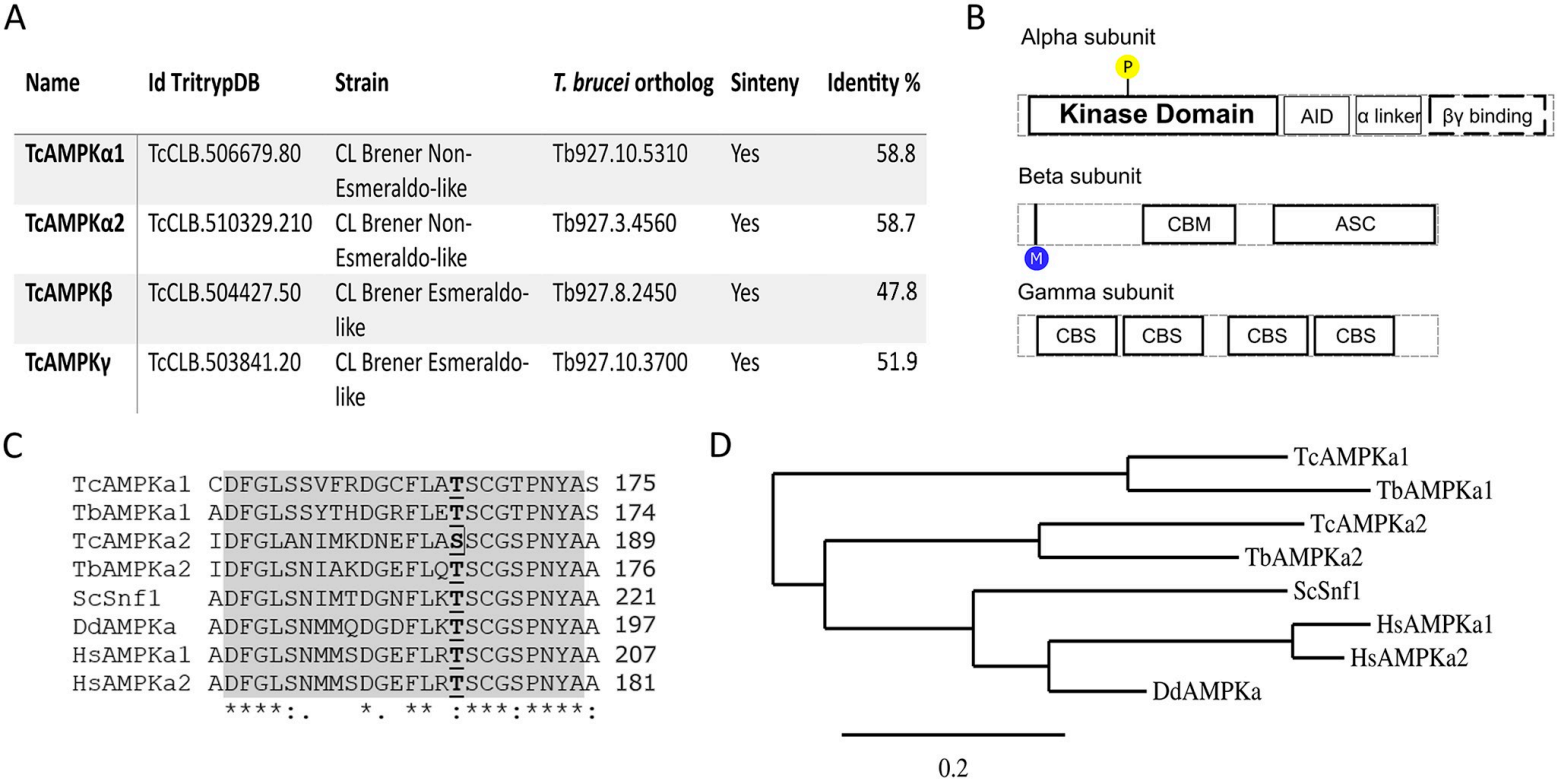
AMPK is partially conserved in *T*. *cruzi*. (**A**) Table of IDs for TcAMPK subunits and the percentage of identity with respect to their orthologous in *T*. *brucei*. (**B**) Protein domains of every TcAMPK subunit. Domains with a dark border line are conserved in *T*. *cruzi*, while domains with dashed lines borders are only partially conserved. AID and α linker domain could not be identified in *T*. *cruzi* catalytic subunits. Domain abbreviations: autoinhibitory domain (AID), carbohydrate-binding module (CBM), association with the SNF1 complex (ASC), cystathionine beta-synthase (CBS). (**C**) Section of the multiple alignment of the alpha subunits of several eukaryotic organisms (*Homo sapiens*, *Saccharomyces cerevisiae*, *Dictyostelium discoideum*, *Trypanosoma brucei* and *Trypanosoma cruzi*). The activation loop conserved region is painted in grey, and the Threonine (or Serine) residue phosphorylated during AMPK activation is in bold font and underlined. (**D**) Phylogenetic tree of the full-length multiple sequence alignment in C. The evolutionary history was inferred using the Neighbor-Joining method [33]. The tree is drawn to scale, with branch lengths in the same units as those of the evolutionary distances used to infer the phylogenetic tree. The evolutionary distances were computed using the JTT matrix-based method [34] and are in the units of the number of amino acid substitutions per site. This analysis involved 8 amino acid sequences. All positions containing gaps and missing data were eliminated (complete deletion option). There were a total of 521 positions in the final dataset. Evolutionary analyses were conducted in MEGA X [35].

Previously, Clemmens et. al. identified the beta and gamma AMPK subunits in *T*. *brucei*, and Salvidia et. al. identified both isoforms of the alpha subunits in the same organism [17,18]. We used those protein sequences as baits for a BLAST search at the Tritrypdb database (https://tritrypdb.org/tritrypdb/) to find the corresponding orthologs in *T*. *cruzi*. The protein sequences retrieved from the CL Brener Esmeraldo like strain for the beta and gamma subunits (IDs TcCLB.504427.50 for the TcAMPKβ and TcCLB.503841.20 for the TcAMPKγ) have 47.78% and 51.92% sequence identity with *T*. *brucei*, respectively. The alpha subunit orthologous genes in *T*. *cruzi* (TcCLB.506679.80 for TcAMPKα1 and TcCLB.510329.210 for TcAMPKα2) have around 58% sequence identity with their respective *T*. *brucei* orthologs (Fig 1A). Interestingly, the paralogs only share around 30% identity between them.

The regulatory subunits candidates show predicted molecular weights (according to their amino acid composition) of 34.38 kDa for the beta subunit and 54.83 kDa for the gamma subunit. In addition, these regulatory subunits contain the protein domains required to function in an AMPK complex (Fig 1B). Key amino acids for the subunits interactions remain conserved, and the protein domains are readily recognized by the NCBI Conserved Domain Search. The TcAMPKα subunits present predicted molecular weight of 81 kDa for TcAMPKα1 and 71 kDa for TcAMPKα2. However, the only conserved region predicted is the kinase catalytic domain in the N-terminal. Several works identified regulatory domains in the C-terminal region of the alpha subunit’s sequence, such as an autoinhibitory domain and interaction domains [30–32]. These C-terminal domains could not be recognized by any informatic tool on the TcAMPKα orthologues, although PROSITE could identify a sequence called “Kinase Associated domain”, of unspecified function. Another relevant trait of the alpha subunit in this kinase complex is the activation loop, which contains a conserved Threonine residue commonly known as Thr172, for its location in the *Rattus sp*. AMPKα protein sequence. This Thr must be phosphorylated to reach maximum levels of kinase activity. As an outstanding feature, TcAMPKα2 presents a Serine residue replacing the Threonine present at the activation loop, further emphasizing the differences between TcAMPKα1 and TcAMPKα2 subunits (Fig 1C). This phosphorylatable residue is highly conserved and TcAMPKα2 is the only coding sequence, to our knowledge, that presents this divergence.

Interestingly, in both *T*. *cruzi* and *T*. *brucei*, AMPKα subunits share a low sequence identity between its paralogous genes, which is surpassed by the identity between orthologous sequences. This could mean that the gene duplication event occurred before the speciation of these two trypanosomatids, illustrated in the phylogenetic tree in Fig 1D. Future studies on the evolution of these genes could uncover neofunctionalization of these AMPK complexes in trypanosomatids.

### Functional complementation in yeast

**Fig 2:**
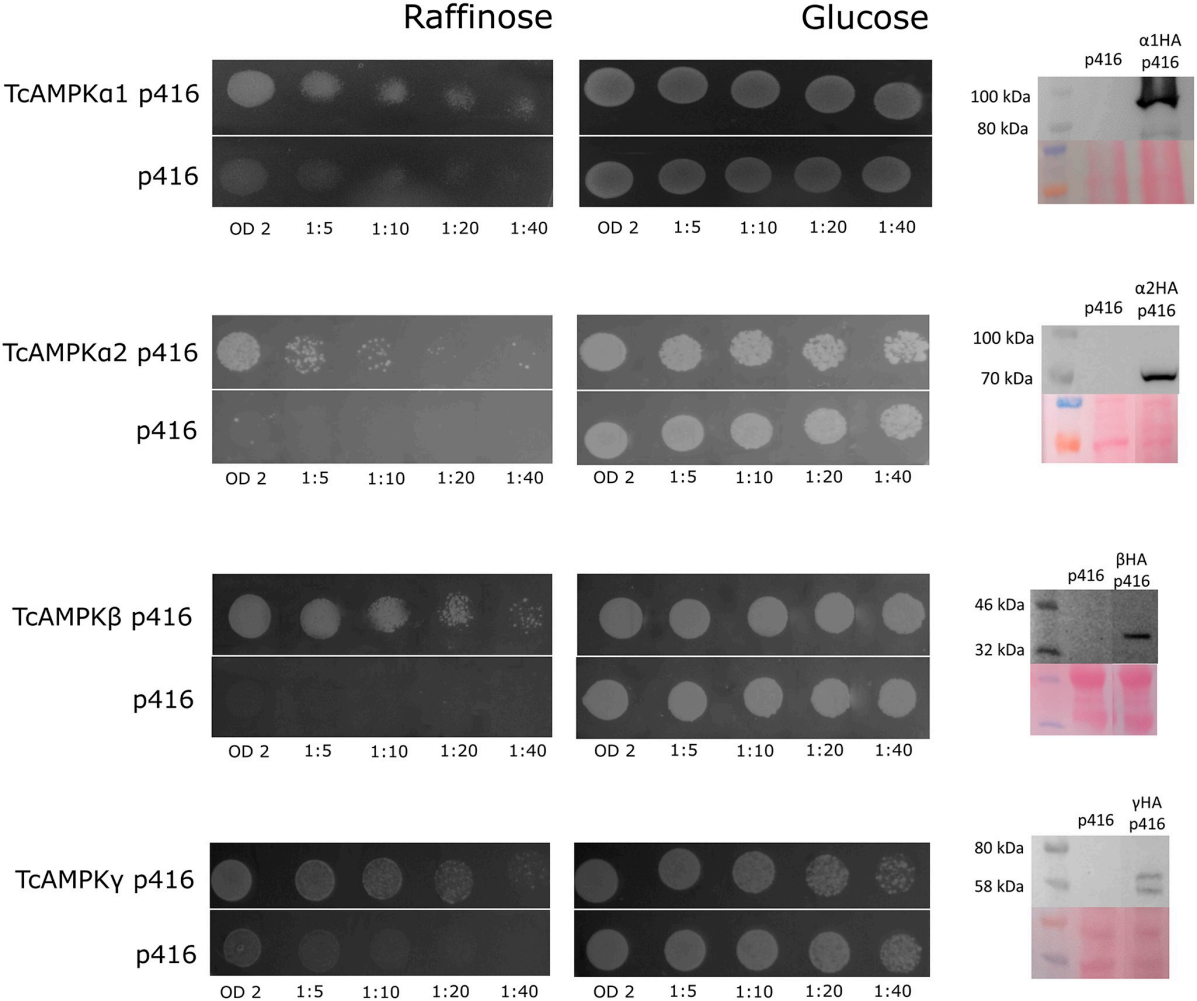
TcAMPK subunits can complement *S*. *cerevisiae* conditional mutants deficient in SNF1 subunits. Transformed yeast were plated in serial dilutions on minimal medium containing an Uracil drop-out and either glucose or raffinose as the only carbon source. Transformation of the conditional mutant yeasts with TcAMPK subunits restored the wild type phenotype, enabling the consumption of raffinose as a carbon source. The yeast containing the empty p416 plasmid could not grow on medium containing raffinose. Western blots at the right margin show at the upper panels the expression of the heterologous construct and bottom panels correspond to Ponceau staining as loading controls.

To evaluate the functional capability of each of the putative TcAMPK subunits, we performed complementation assays in *S*. *cerevisiae* conditional mutant strains, which are alternatively deficient for alpha subunit (MSY1217), the three beta subunits (MSY557) or gamma subunit (MSY846). These mutants are unable to grow in media containing any carbon source other than glucose. The putative TcAMPK subunits were subcloned into p416 yeast expression vector as fusion proteins to a C-terminal HA-tag and used to transform the corresponding yeast strain. All TcAMPK subunits, TcAMPKα1-HA, TcAMPKα2-HA, TcAMPKβ-HA and TcAMPKγ-HA, were able to restore the capability of their specific conditional mutant to use raffinose as a carbon source (Fig 2). On the other hand, the same strains transformed with the empty vector only grew when glucose was added to the culture media, but not when it was replaced by raffinose. These results not only indicate that *T*. *cruzi* AMPK subunits are functional in yeast but also allow us to propose that *T*. *cruzi* AMPK conserves the main subunit functions present in *S*. *cerevisiae*. Western blots of protein extracts from transformed yeasts, revealed with anti-HA antibody, were performed to confirm the expression of each constructs (Fig 2, right panels).

### Evaluation of protein kinase activity in *T*. *cruzi* epimastigotes

**Fig 3:**
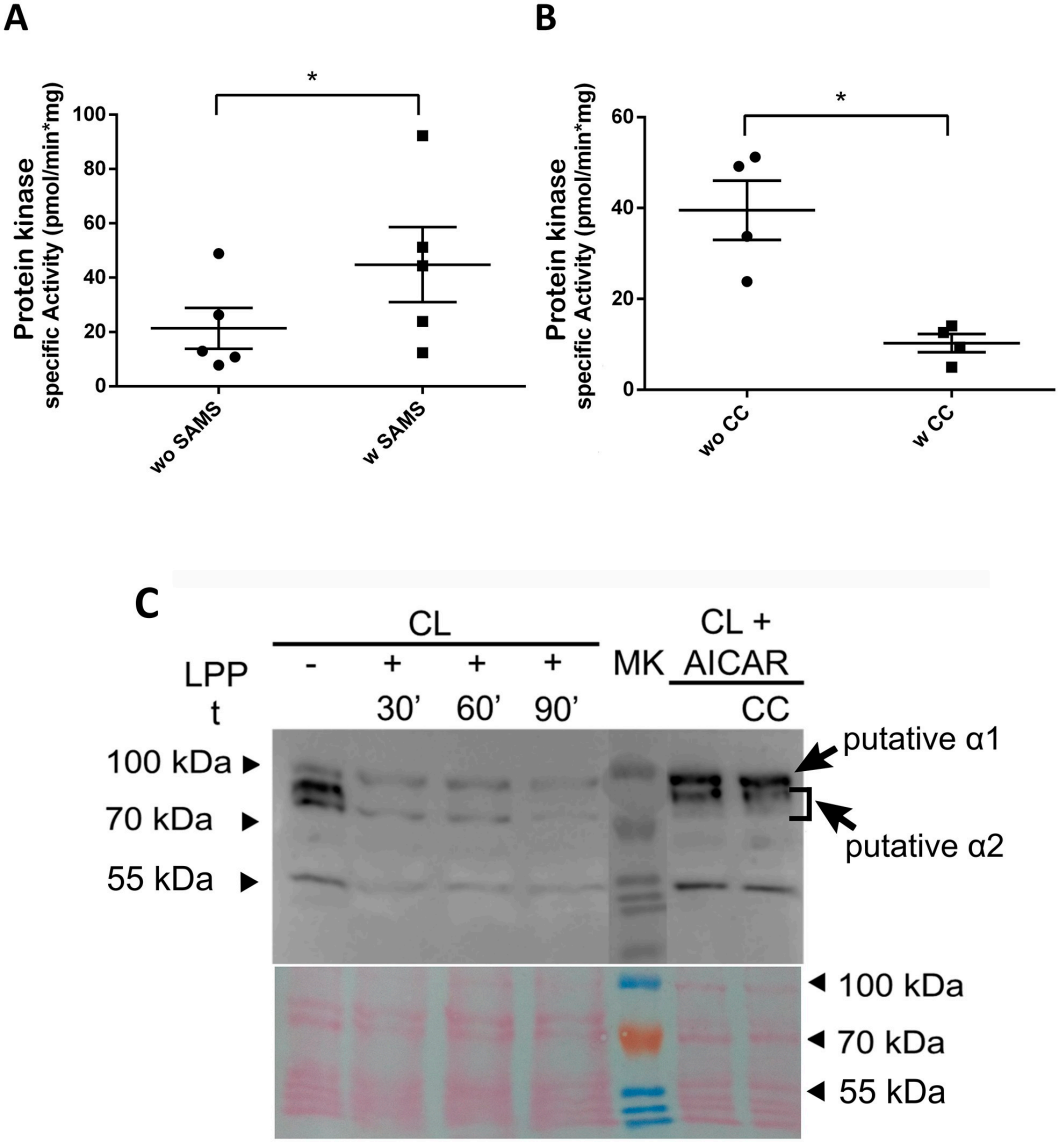
Kinase activity assays and detection of AMPKα subunit phosphorylation confirm *T*. *cruzi* epimastigotes express a functional AMPK. (**A**) Epimastigote whole protein extracts were tested for AMPK substrate, SAMS, phosphorylation by incorporation of ^32^P. Results show a significant increase in activity when SAMS is added to the mix (n = 5). (**B**) Addition of Compound C, an AMPK inhibitor, to the activity mix decreases activity to basal levels (n = 4). (**C**) Phosphorylation of the activation loop of AMPKα subunits was tested with Phospho-AMPKα (Thr172) antibody. Two bands of the expected weights for both TcAMPKα subunits were revealed. Lambda Phage Phosphatase treatment eliminated or reduced these markings. AICAR treatment, a specific AMPK activator, of epimastigotes cultures modified the intensities of the two bands. This effect was not altered by CC simultaneous treatment. All lanes contain equal mass of protein extract. Arrowheads indicate the position of the 55, 70 and 100 kDa marker bands. Bottom panel shows the Ponceau staining as loading control. Representative assay from at least two experiments. CL: CL Brener epimastigote protein extract; LPP: Lambda Phage Phosphatase; MK: molecular weight marker; CC: Compound C; t: time. Error bars represent Standard Error of the Mean. Statistical test corresponds to paired t-test. * p value < 0.05.

Once we determined that TcAMPK subunits can act as functional components of an AMPK complex, capable of restoring the use of raffinose as a carbon source in conditional mutant yeasts, we decided to study the *in vivo* modulation of TcAMPK catalytic activity and the SAMS specific phosphorylation pattern under different nutritional conditions. To begin with, we established the assay conditions to selectively measure AMPK-related kinase (ARK) activity in epimastigote protein extracts by phosphotransference of ^32^P from [γ-^32^P]ATP to the AMPK-preferential substrate peptide, SAMS [36,37]. Fig 3A shows that, under the set assay conditions, without the addition of SAMS in the reaction mix, only a basal kinase activity was observed. On the other hand, when SAMS was added as substrate, kinase activity increased significantly. As a further evaluation of the presence of a phosphotrasferase activity compatible with AMPK activity, we tested the effect of Dorsomorphin (also known as Compound C or CC, a reversible and ATP-competitive inhibitor of AMPK) [38] on the SAMS phosphorylation capability of epimastigotes extract. Fig 3B shows a potent inhibitory effect of CC on the kinase activity, decreasing to basal levels similar to the activity observed in the absence of SAMS.

Another tool that allows the visualization of the activation status of AMPK catalytic subunits is a commercial antibody developed against the phosphorylated state of the Thr172 in the activation loop. We tested this antibody on epimastigote protein extracts and obtained three reaction bands between 70 and 100 kDa of molecular weight (Fig 3C), where the two lower molecular weight bands represent the putative alpha 2 subunit and the upper band represents the putative alpha 1 subunit (see below “Effect of TcAMPKα isoforms overexpression on epimastigotes proliferation”). In addition, a reaction band of approximately 55 kDa is observed, which could be a degradation product of one of the alpha subunits containing the phosphorylated residue. To further confirm their phosphorylated status, we treated the protein extracts with Lambda Phage serine/threonine Phosphatase (LPP) for increasing time periods (30, 60 and 90 min). Throughout these incubation times, it was possible to observe that the bands´ intensity decreased, where the upper band of the putative TcAMPKα2 subunit becomes undetectable after the first 30 min of treatment with LPP. We also studied the AMPK phosphorylation state in response to specific treatments with AMPK activity modulators. We treated epimastigotes with AICAR, an adenosine analog which is known to act as a specific AMPK activator after it is converted inside the mammalian cells to ZMP, an AMP analog [39], and observed an increase in the intensity of the putative TcAMPKα1 subunit (upper band, CL + AICAR) and a decrease in the intensity of the putative TcAMPKα2 lower band, in comparison with the control lane (CL -). Incubation with both AICAR and CC didn’t change the pattern observed in CL + AICAR. These results can be explained by the mechanisms of action of the two compounds. AICAR is an analog of AMP and, as such, it can activate AMPK by binding with the gamma subunit, inducing a conformational change that protects the Thr172 from dephosphorylation, while CC is a competitive inhibitor binding to the ATP site in the catalytic subunit. Hence, CC inhibits the kinase activity but doesn’t necessarily affect the phosphorylation status.

Gel regions between the 70kDa—100kDa molecular weight of denaturing SDS-PAGE of protein extracts were excised and prepared for mass spectrometry analysis. Through this analysis, we have been able to detect the endogenous expression of TcAMPKα2 and its phosphorylation in the Serine replacing the more canonical Threonine in the activation loop (S1 Fig). This result confirms the possibility of a catalytic subunit activation with a natural Ser for Thr substitution in an AMPK catalytic subunit.

### Catalytic activation of TcAMPK under nutritional stress

**Fig 4:**
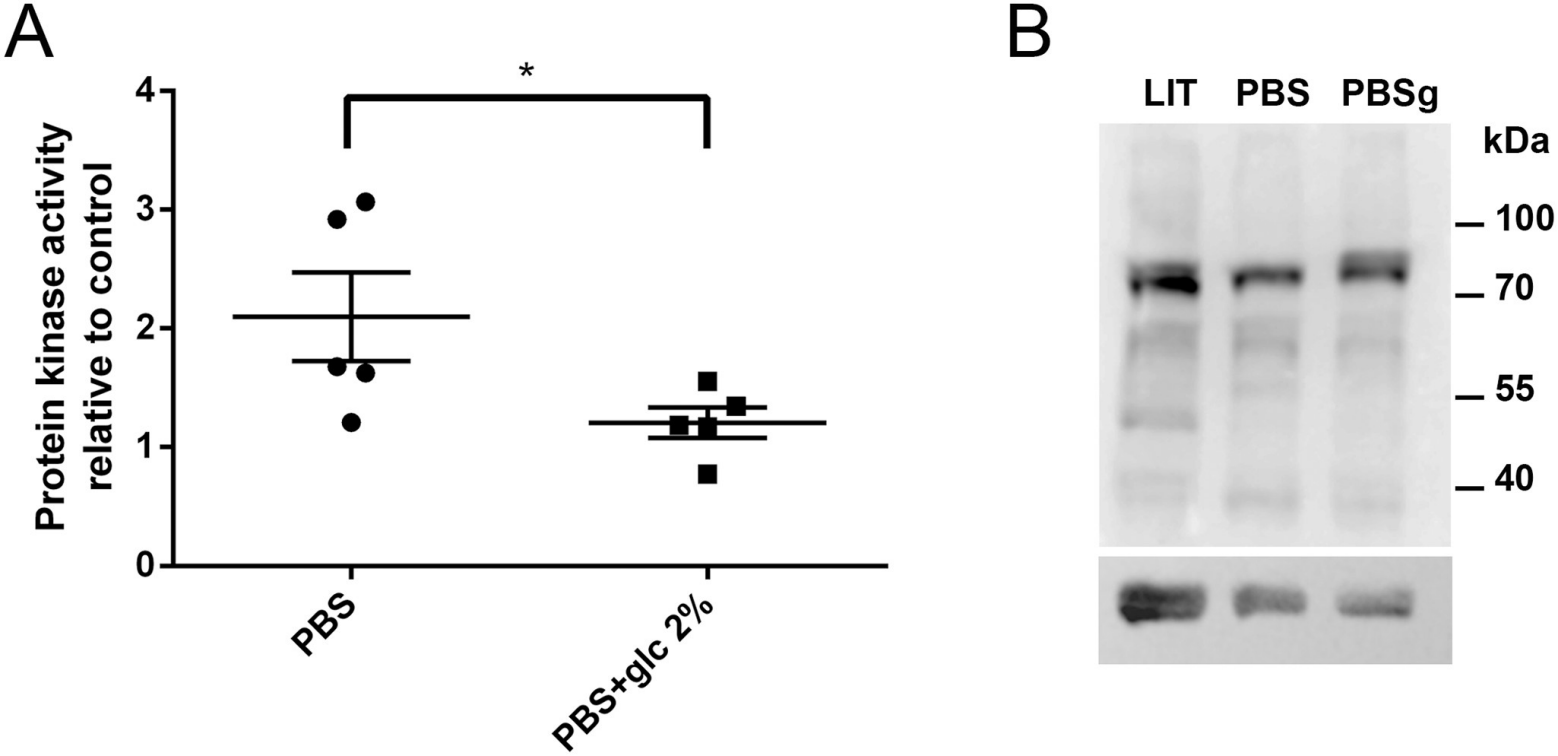
Nutritional stress increases AMPK activity in epimastigotes, and addition of glucose keeps AMPK activity at basal levels. (**A**) Protein kinase activity was tested in the presence of SAMS and epimastigote whole protein extracts. Epimastigotes were incubated for 17 hs alternatively in LIT medium as control, PBS or PBS containing 2% (w/v) glucose. Measurements were normalized to LIT cultures and a paired t-test was performed to compare AMPK activity differences (n = 5) *p value < 0.05. (**B**) Western blot of epimastigote protein extracts under these same treatments revealed with the anti-Phospho AMPKα antibody. Bottom panel shows the same membrane revealed with anti-α tubulin antibody as loading control (~55 kDa).

In many eukaryotic organisms, from yeast (SNF1 kinase, a homologue of AMPK) to plants (SnRK1, a homologue of AMPK) to humans, AMPK is a metabolic regulator that is activated when energy metabolites (lower ATP levels and increased intracellular AMP concentration) and nutrients (carbon source) are limited [40]. Following this rationale, we hypothesized that TcAMPK could be a key enzyme sensing the different metabolic states that epimastigotes undergo during its passage through the insect gut. To further evaluate this hypothesis, *T*. *cruzi* epimastigotes were exposed to nutritional stress by incubation in Phosphate Buffered Saline (PBS) during seventeen hours at 28°C. This condition has previously been used to study other processes related to nutritional stress in these parasites, such as autophagy [41,42]. The ARK catalytic activity after this treatment showed a mean 2-fold increase over the activity in the control condition. On the other hand, incubation in the same buffer supplemented with glucose 2% (w/v) prevented the enzyme activity increase. Protein kinase activity values were normalized to those obtained from parasites grown in LIT medium (Fig 4A). These results are in turn strengthened through western blot assays using the anti-Phospho AMPKα antibody under the same starving conditions in PBS or in the same buffer with glucose. In Fig 4B, a differential phosphorylation pattern can be observed under starving conditions (PBS) when compared with LIT or PBSg. A consistent double band pattern is observed in LIT and PBSg, while in the absence of carbon sources (PBS) a single band is observed. Overall, these observations indicate that TcAMPK alpha subunits can change their phosphorylation state by the absence of a carbon source and suggest a possible role initiating metabolic responses to face prolonged nutritional stress in *T*. *cruzi* epimastigotes.

### Effect of TcAMPKα isoforms overexpression on epimastigotes proliferation

**Fig 5:**
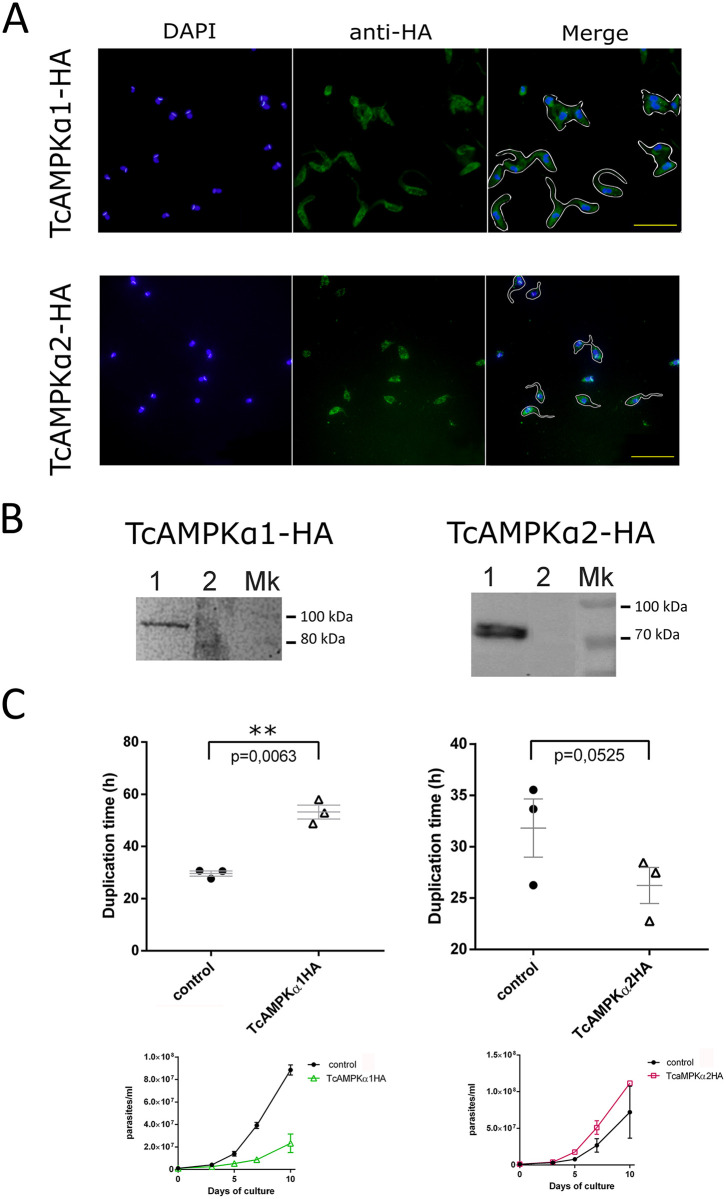
Over-expression of TcAMPKα isoforms in epimastigotes leads to antagonistic effects in proliferation. (**A**) Immunofluorescence images showing the cytoplasmic expression of the constructs. In the Merge quadrants some cells were contoured to illustrate the morphology of the parasites. Scale bar = 50 μm. (B) (**B**) Western blot of protein extracts of epimastigotes showing the correct expression of the TcAMPKα1 and TcAMPKα2 subunits tagged to HA. Lanes 1 of both gels show the corresponding subunit detected by the anti-HA antibody and lanes 2 correspond to a negative control. (**C**) Upper panels show doubling times of epimastigote cultures during the exponential phase of the growth curve (between day 3 and day 7) (n = 3). Control corresponds to epimastigotes transfected with the empty pRIBOTEX vector. Error bars represent the Standard Error of the Mean and comparison was analyzed by a t-test. ** p value < 0.01. Lower panels show growth curves of epimastigotes cultures (n = 3). Control corresponds to CL Brener wild type epimastigotes. Error bars represent the Standard Error of the Mean. Epimastigotes transfected with empty pRIBOTEX vector and CL Brener wild type cells show no difference in growth patterns.

To further reveal metabolic pathways that involve TcAMPK, both TcAMPKα1-HA and TcAMPKα2-HA constructs were subcloned into the pRIBOTEX expression vector and CL Brener *T*. *cruzi* epimastigotes were then independently transfected with these plasmids or with the empty vector. After the selection of stable transgenic lines, the expression and intracellular localization of these two proteins were investigated by western blot and indirect immunofluorescence using an anti-HA antibody (Fig 5A and 5B). Western blots showed both overexpressed proteins of molecular weights between 70 kDa and 100 kDa, with TcAMPKα1-HA slightly heavier than TcAMPKα2-HA (Fig 5B). Interestingly, TcAMPKα1-HA appeared as a single band, while TcAMPKα2-HA appeared as two bands of very similar weight. This is in agreement with what we could observe on wild type epimastigotes extracts using an anti- phosphorylated Thr172 antibody (Fig 3C). This double band could suggest a post-translational modification affecting the protein migration. Further assays will be necessary to reveal the nature of this modification.

Cellular localization was similar for both proteins: distributed in the cytosol in a granulated pattern, which could point to a partial association with small organelles such as glycosomes or acidocalcisomes (Fig 5A). Parasites transfected with the empty vector or non-tagged construct didn’t show any fluorescent signal when revealed with anti-HA antibody.

We assessed the growth curves of the overexpressing cultures in regular conditions. Epimastigotes were counted in a Neubauer chamber for up to 10 days after initial dilution. Quantifications made between day 3 and 7 were considered as the exponential phase to evaluate the doubling time. Results show that overexpression of each isoform of the catalytic subunit caused opposite effects on proliferation (Fig 5C). Overexpression of TcAMPKα1-HA presents deleterious effects on epimastigotes, increasing doubling time in a statistically significant manner. These cultures didn’t reach maximum density and eventually arrested proliferation and died. Overexpression of TcAMPKα2-HA, instead, led to a slight decrease in doubling time during the exponential phase of the growth curve. These epimastigotes didn’t show any other effect on phenotype and reached normally the maximum density.

These results show that the overexpression of the TcAMPKα subunits produces antagonistic effects on the proliferation of epimastigotes.

### Correlation between TcAMPK expression and autophagic response in epimastigote cells

Since AMPK is involved in autophagy in several eukaryotic organisms in response to different kind of stresses, and considering the essential role of autophagy in the progression of *T*. *cruzi* life cycle, we sought to investigate if TcAMPK is capable of modulating autophagosome formation. To this end, we used Monodansylcadaverine (MDC), an acidotropic fluorescent dye that binds specifically to autophagosome membranes. After starving epimastigotes for 17 h in PBS, we measured MDC incorporation. We evaluated this parameter in wild type epimastigotes and the TcAMPKα2-HA overexpressing line.

**Fig 6:**
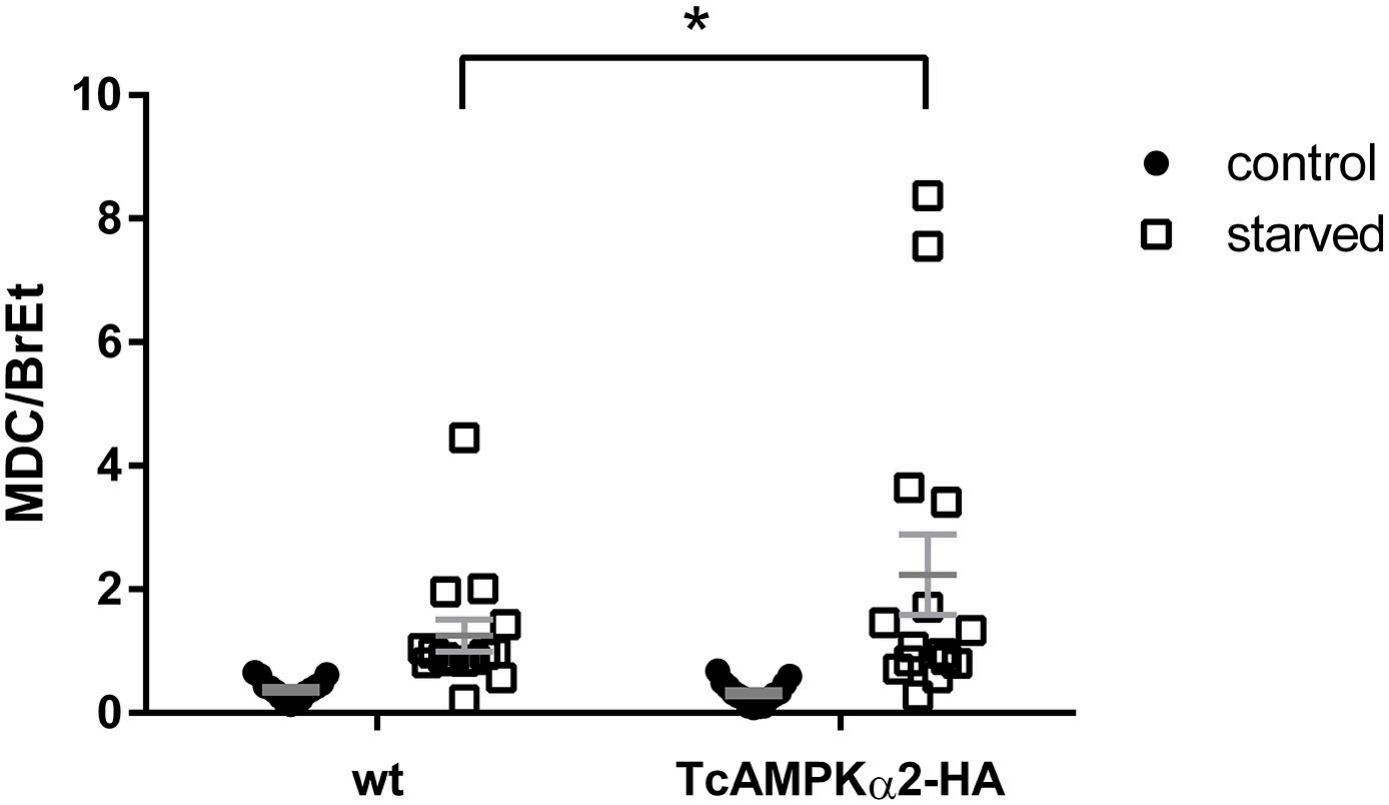
TcAMPKα2 overexpressing epimastigotes have an increased autophagic capacity. MDC incorporation was measured for wild type and overexpressing epimastigotes after 17 h incubation in either LIT medium (control) or PBS (starved). MDC values were normalized to Ethidium Bromide (BrEt) incorporation. MDC/BrEt ratio is expressed in arbitrary units. A t-test analysis of the starvation condition between the two strains shows a significant difference (n = 15). There were no significant differences in the control conditions. Error bars represent the Standard Error of the Mean.

Our results show that the overexpression of TcAMPKα2 led to a higher autophagic capacity (Fig 6). The overexpressing epimastigotes can reach higher levels of MDC incorporation. Although marked inter-assay variations were observed, higher levels of MDC incorporation could always be registered in overexpressing parasites compared to WT ones. The intrinsic variations of the assay could be explained by the cyclic nature of autophagy itself, which will be further discussed in the Discussion section.

## Discussion

*Trypanosoma cruzi* needs to precisely control energy homeostasis, nutrient availability, proliferation and differentiation in order to respond to changes in the environment and simultaneously preserve the viability of the host and the permanence of the disease. Some protein complexes act as hubs in the regulation of cell responses to environmental queues, the AMPK being one of them. Here we report, for the first time, the presence of two different AMPK complexes in *T*. *cruzi* and describe novel functions for TcAMPK as a key factor in the regulation of autophagy and proliferation, leading to a link between nutrient sensing, cell cycle and stage differentiation.

Our results show that *T*. *cruzi* genome contains coding genes for at least two different AMPK complexes. Epimastigotes express a kinase activity specific for an AMPK substrate, which is endogenously regulated, as shown by Thr172 phosphorylation and protein kinase activity under different treatments.

Yeast complementation exposed that every *T*. *cruzi* subunit has the necessary protein domains to function as part of an AMPK complex. Previous studies have shown that alpha subunits that cannot interact with the AMPK regulatory subunits, could retain a basal protein kinase activity [6]. Therefore, for these *T*. *cruzi* subunits, we could only establish a Snf1 function in yeast that restores their ability to use alternative carbon sources, although we cannot confirm glucose repression or interaction with the regulatory subunits. It is yet to be confirmed that all *T*. *cruzi* AMPK subunits interact with each other forming a heterotrimeric complex. Regarding this last concern, Saldivia et. al. had already demonstrated by co-immunoprecipitations, that both TbAMPK complexes, containing α1 or α2 subunits, are expressed in the bloodstream stage [18].

It is well known that AMPK in other eukaryotic organisms is regulated both allosterically and by post-translational modifications [43]. The best defined mechanisms of AMPK activation are the phosphorylation at Thr172 of the α-subunit and by AMP and/or adenosine diphosphate (ADP) binding to γ-subunit [44]. Here, we demonstrated that TcAMPK could be regulated by phosphorylation at the activation loop. Western blots using the Phospho-AMPKα (Thr172) antibody show three bands in epimastigotes extracts, with sizes compatible with those of their theoretical molecular weights, which are also similar to the weight of the *T*. *brucei* catalytic subunits. Integrating this result with those obtained when alpha subunits were independently overexpressed in epimastigotes, we concluded that the upper band (with less electrophoretic mobility) corresponds to TcAMPKα1 subunit and the lower two bands correspond to TcAMPKα2 subunit. Another outstanding feature of the TcAMPKα2 subunit is the presence of a phosphorylatable Serine, instead of a Threonine, in the activation loop. To our knowledge, this is the only organism presenting this characteristic, and we hypothesize that it could differentially influence the upstream regulation of the complexes containing this subunit. The phosphorylation pattern of TcAMPKα subunits can be modified by nutritional stress and treatment with AMPK activators. These results point to the existence of at least one heterotrimeric complex in *T*. *cruzi*, which is able to sense AMP analogs and be activated by phosphorylation at the activation loop (in both alpha subunits) in consequence. According to our results shown in Fig 4B, the band identified as the putative TcAMPKα1 shows the major phosphorylation pattern changes when parasites are subjected to starvation and it is possible that this catalytic subunit is responsible for sensing nutritional conditions.

The kinase activity assays allowed us to conclude that epimastigotes express an AMPK-related kinase activity, which is affected by the availability of glucose in the medium. Some studies report that other kinases present different levels of specificity for SAMS and Compound C [45,46]. We realize that ARKs could phosphorylate SAMS or be inhibited by Compound C in a similar way as AMPK. However, we are confident that the effect we observe on ARK enzymatic activity is due to an AMPK activity difference, as is suggested by the modification in the phosphorylation pattern, when revealed by Anti-phosphoAMPK antibody. A more thorough understanding of the regulation of AMPK in *T*. *cruzi* could be achieved by immunoprecipitation of the complex and evaluation of allosteric regulation *in vitro*.

To assess the relative influence of AMPK activation on parasite metabolism, we used epimastigotes of *T*. *cruzi* overexpressing each of the catalytic subunits and found that this resulted in phenotypes associated with proliferation and nutritional stress response. Interestingly, these over-expressions had antagonistic effects on proliferation progression. TcAMPKα1 over-expression hindered proliferation, resulting in longer doubling times and in the inability to reach maximum parasite density, leading to eventual loss of the cultures. Overexpressing the enzyme could be detrimental for the progression of the cell cycle due to induced metabolic stress, leading to a slower proliferation. Other possibility suggests the overexpression could be affecting cell cycle progression directly, leading to an arrest effect in proliferation. This effect could be linked to an induction of the differentiation process (metacyclogenesis) that occurs in the final compartment of the intestinal tract (the posterior region of the small intestine and the rectum) [47]. Saldivia et. al. already showed that the TbAMPK complex containing the α1 subunit is involved in the process of differentiation from the slender to the stumpy bloodstream form [18]. Over the years, several factors have been implicated to influence metacyclogenesis, such as osmolarity [48], the initial pH of the media [49], the carbon source availability [50] and more recently, autophagy [41,51,52]. However, the molecular bases of the morphogenetic alterations necessary and sufficient to elicit parasite differentiation remain to be fully elucidated. It would be interesting, through other techniques such as the use of inducible vectors, to assess how the constitutive overexpression of TcAMPKα1 causes the parasites to divide more slowly resulting in non-viable cell cultures. TcAMPKα1 also showed interesting phylogenetic characteristics, raising the possibility of a neofunctionalization through evolution of these parasites.

On the other hand, TcAMPKα2 over-expression led to shorter doubling times during the exponential growth phase. In other species, AMPK complexes have antagonistic functions even with fewer amino acid sequence differences. This was also observed in *H*. *sapiens*, where AMPKα1 subunit has been classified as oncogene, because of its higher expression in some types of cancer cells, while AMPKα2 seems to function as a tumor suppressor instead [53]. The authors state that these differences in function cannot be fully explained by differences in substrate specificity but are rather determined by residues modified by regulatory upstream proteins, affecting AMPK activity and localization. In *T*. *cruzi*, sequence differences are more extensive between alpha subunits, and it would be important to evaluate if this affects substrate specificity and how, in addition to its regulation. Furthermore, epimastigotes over-expressing TcAMPKα2 were able to reach higher autophagic activity during prolonged nutritional stress, in comparison with control lines. Autophagy is an essential process in the progression of the life cycle of trypanosomatids [41,54,55] and AMPK is known to be involved in these processes in an ancestral and conserved manner [21,22,56–59]. We hypothesize that TcAMPKα2 would induce the autophagy process to cope with the nutritional stress caused by the absence of glucose as an energy source. This is supported by an increase in ARK activity in epimastigotes in the absence of glucose. The fact that TcAMPKα2 overexpression leads to an acceleration in cell duplication during the exponential phase can be interpreted as a role of the TcAMPKα2 subunit in the rapid consumption of glucose and maintenance of proliferation while there is availability of this carbon source, similarly to what would happen right after the insect vector takes a blood meal. After the insect hasn’t fed in several weeks, amino acids are the most abundant carbon source in the midgut and parasite density increases significantly, which leads to an arrest in proliferation. These results point to the presence of two AMPK complexes with distinct functions, although their exact roles in the parasite life cycle are yet to be determined.

Additionally to the role of AMPK complexes in the rapid response to stress through the phosphorylation of downstream effectors, they also modulate gene expression by phosphorylating transcription factors [60]. In kinetoplastids, gene transcription is poorly modulated, there are no promoter sequences and transcription is carried out as polycistronic units processed by trans-splicing [61]. mRNA translation is then regulated by modifying their stability, localization and translationality. For that reason, RNA Binding Proteins (RBPs) and RNA regulons have shown to have essential roles in different cellular processes in these organisms, such as differentiation and infectivity [62,63], and responses to nutrient and oxygen availability [64,65]. One of our hypotheses regarding new roles for the AMPK complexes in trypanosomatids involves its interaction with the RBPs and influencing regulon activation. Our preliminary bioinformatic searches have shown some RBPs in *T*. *cruzi* and *T*. *brucei* that present the conserved sequences for AMPK phosphorylation, which makes them potential AMPK substrates. Furthermore, Saldivia et. al. identified the *T*. *brucei* RBP27 as an interactor of one of the TbAMPK complexes when co-immunoprecipitated. AMPK could function as the ancestral link between environmental conditions sensing and rapid and long-term cellular response through these RBPs and other novel substrates.

To the extent of our knowledge this work brings the first evidence on a role for the AMPK complex in trypanosomatids during nutritional stress response. Our results suggest that the novel AMPK complex present in trypanosomatids was generated by AMPKα gene duplication in a common ancestor, preceding speciation, even though more experimental data must be generated to confirm this observation.

The finding in *T*. *cruzi* of a novel AMPK complex, which evolved incorporating unique characteristics, points out this pathway as a possible chemotherapy target for Chagas’ disease. Combining the background knowledge on AMPK complexes and the available compound libraries to affect its enzymatic activity, TcAMPK complexes are also promising targets for therapeutic drug repositioning.

## Supporting information

S1 Fig Mass spectrometry analysis of epimastigotes protein extract to detect TcAMPKα subunits endogenous expression and its phosphorylation. (**A**) The central column of the table depicts the sequence of the peptide constructed from the protein fragments detected. This peptide corresponds to the activation loop of the TcAMPKα2, with a Serine in the position of the more conventional Thr172. Red and Blue numbers on the table are molecular weights of the peptides detected in the mass spectrometer, while black numbers are obtained by digital simulation of the protein digestion by the same proteases. (**B**) Spectra of the peptide sequence reconstructed in A. In the green circle is the peptide providing proof of the Serine phosphorylation.
(PDF)
