# Supplementary material for: An AMP-activated protein kinase complex with two distinctive alpha subunits is involved in nutritional stress responses in Trypanosoma cruzi
Source: PLoS Negl Trop Dis. 2021 May 24;15(5):e0009435. doi: 10.1371/journal.pntd.0009435 (PMC8177656; doi:10.1371/journal.pntd.0009435)

Supplementary Figure 1

Mass spectrometry analysis of epimastigotes protein extract to detect TcAMPKα subunits endogenous expression and its phosphorylation. (A) The central column of the table depicts the sequence of the peptide constructed from the protein fragments detected. This peptide corresponds to the activation loop of the TcAMPKα2, with a Serine in the position of the more conventional Thr172. Red and Blue numbers on the table are molecular weights of the peptides detected in the mass spectrometer, while black numbers are obtained by digital simulation of the protein digestion by the same proteases. (B) Spectra of the peptide sequence reconstructed in A. In the green circle is the peptide providing proof of the Serine phosphorylation.

A

#1	b^{+}	b^{2+}	b^{3+}	Seq.	y^{+}	y^{2+}	y^{3+}	#2
1	116.03422	58.52075	39.34959	D				23
2	230.07715	115.54221	77.36390	N	2392.05267	1196.52997	798.02241	22
3	359.11974	180.06351	120.37810	E	2278.00974	1139.50851	760.00810	21
4	506.18815	253.59772	169.40090	F	2148.96715	1074.98721	716.99390	20
5	619.27222	310.13975	207.09559	L	2001.89873	1001.45301	667.97110	19
6	690.30933	345.65830	230.77463	A	1888.81467	944.91097	630.27641	18
7	857.30769	429.15748	286.44075	S-Phospho	1817.77756	909.39242	606.59737	17
8	944.33972	472.67350	315.45142	S	1650.77920	825.89324	550.93125	16
9	1104.37037	552.68882	368.79497	Carbamidometl	1563.74717	782.37722	521.92057	15
10	1161.39183	581.19955	387.80213	G	1403.71652	702.36190	468.57702	14
11	1248.42386	624.71557	416.81280	S	1346.69506	673.85117	449.56987	13
12	1345.47662	673.24195	449.16373	P	1259.66303	630.33515	420.55919	12
13	1459.51955	730.26341	487.17803	N	1162.61026	581.80877	388.20827	11
14	1622.58288	811.79508	541.53248	Y	1048.56734	524.78731	350.19396	10
15	1693.61999	847.31363	565.21152	A	885.50401	443.25564	295.83952	9
16	1764.65711	882.83219	588.89055	A	814.46689	407.73709	272.16048	8
17	1861.70987	931.35857	621.24147	P	743.42978	372.21853	248.48144	7
18	1990.75246	995.87987	664.25567	E	646.37702	323.69215	216.13052	6
19	2103.83653	1052.42190	701.95036	I	517.33442	259.17085	173.11633	5
20	2216.92059	1108.96393	739.64505	L	404.25036	202.62882	135.42164	4
21	2303.95262	1152.47995	768.65572	S	291.16630	146.08679	97.72695	3
22	2360.97408	1180.99068	787.66288	G	204.13427	102.57077	68.71627	2
23				K	147.11280	74.06004	49.70912	1

B

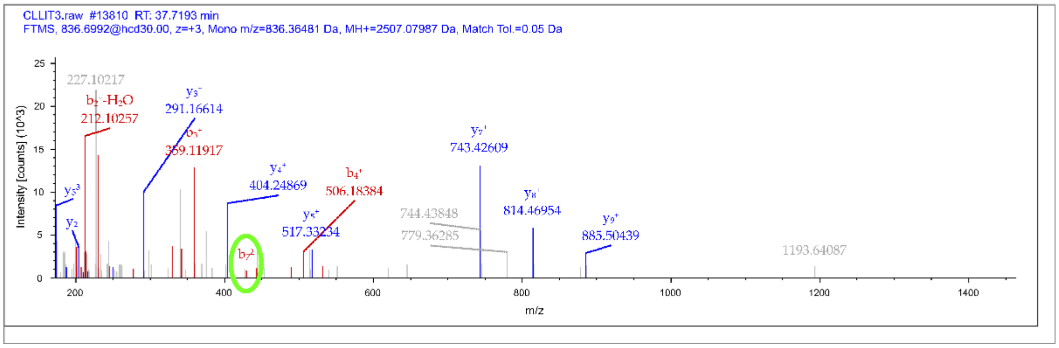

Supplement: S1 Fig — (A) The central column of the table depicts the sequence of the peptide constructed from the protein fragments detected. This peptide corresponds to the activation loop of the TcAMPKα2, with a Serine in the position of the more conventional Thr172. Red and Blue numbers on the table are molecular weights of the peptides detected in the mass spectrometer, while black numbers are obtained by digital simulation of the protein digestion by the same proteases. (B) Spectra of the peptide sequence reconstructed in A. In the green circle is the peptide providing proof of the Serine phosphorylation. (PDF) [file pntd.0009435.s001.pdf]
